# Schöne Steine – und strahlende Gesichter: Ein ungewöhnlicher Einsatz

**DOI:** 10.1007/s00101-023-01282-4

**Published:** 2023-05-24

**Authors:** Marc-Michael Ventzke, Werner Kirchinger

**Affiliations:** 1 Anästhesiologie und Operative Intensivmedizin, Medizinische Fakultät, Universität Augsburg, Stenglinstraße 2, 86156 Augsburg, Deutschland; 2 Regionales Strahlenschutzzentrum Neuherberg, Helmholtz Zentrum München, Ingolstädter Landstraße 1, 85764 Neuherberg, Deutschland

## Anamnese und kalte Lage

Der Inhaber einer Recyclingfirma hatte diverse Kisten unterschiedlichen Inhalts erworben. Bei der Sichtung zu Hause fielen ein Kasten mit dem Strahlenwarnzeichen und der Bezeichnung „Radioaktiv Klasse I“ und mehrere evtl. radioaktiv strahlende Gesteinsbrocken auf. Die integrierte Leitstelle (ILS) entsandte nach „ABC‑2, Gefahrstoff/undefinierbarer Gegenstand“ und dem Zusatz „Uranfund“ um 16:40 Uhr die Feuerwehr (Fw) mit dem Fachberater Gefahrgut und einer CBRN(chemische, biologische, radiologische und nukleare Gefahren)-Messeinheit.

Seitens des Rettungsdienstes (RD) erfolgten die Entsendungen eines Notarzteinsatzfahrzeugs (NEF) und Rettungswagens (RTW) sowie des Einsatzleiter Rettungsdienstes (ELRD).

Die Fw erkundete den Fundort. Da die Ladung auf einem Anhänger war, veranlasste der Einsatzleiter der Fw die Verbringung des Anhängers durch den 41-jährigen Sohn (selbst Mitglied der Fw und Strahlenschutzbeauftragter) auf ein außerhalb der Ortschaft liegendes Feld.

Die Einsatzstelle lag außerhalb an einem Waldrand. Der Gefahrenbereich wurde durch die Fw mit einem Radius von 50 m um den Anhänger festgelegt.

Die Art der Strahlenquellen war nicht bekannt, daher führte die Fw Messungen an der Außenseite des Anhängers und an den Objekten durch (Zusatzmaterial online: Tab. 1). Der Sohn (Patient 1) hatte ebenso wie sein Vater (Patient 2) den Inhalt der Kisten inspiziert.

Es musste von einer Kontamination durch die Handhabung ausgegangen werden, daher wurden beide untersucht.

Hierbei konnte die Fw erhöhte Strahlungswerte im Bereich der Hände und im Gesicht/Kopfbereich bei Patient 1 feststellen (Zusatzmaterial online: Tab. 2).

Um eine weitere Verschleppung zu verhindern, wurde er auf dem Feldweg isoliert und angewiesen, medizinische Untersuchungshandschuhe anzuziehen.

Durch die vor Ort befindlichen Kräfte des RD waren eine medizinische Einordnung der Messwerte, eine Einschätzung über ein sinnvolles weiteres Vorgehen und eine Gefährdungsbeurteilung der Bevölkerung nicht möglich. Sie forderten daher in Absprache mit der Fw die Sanitätseinsatzleitung (leitender Notarzt (LNA) und organisatorische Leiter (OrgL)) nach. Durch Erhöhung des Einsatzstichwortes entsandte die ILS weitere Kräfte des RD (Zusatzmaterial online: Tab. 3).

Der LNA traf um 17:47 Uhr an der Einsatzstelle ein, um mit dem dortigen Abschnittsleiter der Fw eine medizinische Lageerkundung vorzunehmen.

Die restlichen Kräfte befanden sich mit dem OrgL am ca. 2 km entfernten Fw-Haus in Bereitstellung, wo die Einsatzleitung und rückwärtige Führungsstelle eingerichtet waren.

## Befund und Diagnose

Bei Eintreffen an der Einsatzstelle fand der LNA Patient 1 auf dem Boden sitzend vor, bei dem nach kurzer Anamneseerhebung und Rücksprache aktuell keine gesundheitliche Beeinträchtigung vorlag.

Über die Umgebungsstrahlung hinausgehende Strahlenwerte konnten an beiden Händen und im Kopfbereich mit 60 Bq/cm^2^ und 40 Bq/cm^2^ nachgewiesen werden. Eine Inkorporation konnte nicht ausgeschlossen werden. Aufgrund der Messwerte gingen die Rettungskräfte von einer Kontamination mit radioaktiven Partikeln aus.

## Therapie und Verlauf

Nachdem für die Kräfte des RD keine Gefahr bestand, verlegten ELRD, OrgL, NEF und 1 RTW an die Einsatzstelle.

Zusammen mit den vor Ort befindlichen Fachberatern wurden mehrere Möglichkeiten der Dekontamination diskutiert.

Eine Dekontamination mit Wasser (Duschen im Fw-Haus) wurde verworfen, da hierdurch eine Verschleppung sowohl am Patienten als auch örtlich befürchtet wurde. Letztlich wies man den Patienten nach Anleitung an, selbst eine Trockendekontamination mittels Papierhandtüchern und Schnäuzen der Nase durchzuführen (Zusatzmaterial online: Abb. 1). Eine weitere Messung erbrachte keine über den Nulleffekt hinausgehenden Messwerte, sodass von einer erfolgreichen Dekontamination ausgegangen werden konnte.

Der Betroffene wurde ausführlich aufgeklärt.

Der Anhänger mit den Materialien wurde noch vor Ort durch Fachkräfte der Werk-Fw des Helmholtz-Zentrums München untersucht. Hierbei konnte Radium 226 als Zerfallsprodukt von Uran detektiert werden. Auf Anweisung des Bayerischen Landesamtes für Umwelt wurde der Anhänger nach Sicherung durch die Fw zur weiteren Untersuchung nach Augsburg verbracht (Zusatzmaterial online: Abb. 2).

Dort fanden sich Tritium-Leuchtquellen, Glühstrümpfe und Rauchmelder mit radioaktivem Inhalt, daneben eine größere Menge Granitgestein sowie ein Überspannungsschutz mit radioaktiven Ionisationsverstärkern (Zusatzmaterial online: Abb. 3).

Die Spezialuntersuchungen der Ausscheidungsproben (3 Tage Sammelurin) auf eine mögliche Inkorporation ergaben keinerlei Hinweis auf eine Inkorporation.

## Diskussion

Strahlenunfälle sind in Deutschland extrem selten. Beim Stichwort „Strahlenunfall“ werden Assoziationen von apokalyptischen Szenarien wie brennenden Kernkraftwerken und verunfallten Brennelementebehältern geweckt. Viel häufiger dürften jedoch Berührungspunkte der Fw und des RD mit kleinen Strahlenquellen geringer Aktivität sein. Diese finden sich z. B. im Straßenverkehr [1].

Aufgrund der Seltenheit der Ereignisse und des Umgangs mit Strahlenquellen, der „Unsichtbarkeit“ der Strahlung und oft unbekannten Messwerten, Einheiten und deren medizinischer Bedeutung resultieren zumeist erhebliche Unsicherheiten bei Patienten und RD: Wie soll mit den Patienten umgegangen werden? Welche medizinische Auswirkung hat die Strahlenexposition? Ist man selbst als Einsatzkraft gefährdet? Welche persönliche Schutzausrüstung ist sinnvoll?

Einen weitergehenden Überblick über Strahlenunfälle und deren Management im Rettungsdienst gibt der Leitfaden *Der Strahlenunfall* (momentan in Neuauflage) [2] und der darauf basierende Weiterbildungsartikel [3]. Außerdem wurde 2022 die Empfehlung der Strahlenschutzkommission veröffentlicht [4].

Skazel et al. beleuchten in ihrem Übersichtsartikel die Aspekte der Organisation und Weiterversorgung im Krankenhaus sowie das klinische Management von Strahlenverunfallten [5].

Das Vorgehen an der Einsatzstelle orientierte sich an den 5 Regeln des Strahlenschutzes, den sog. 5A [1, 6]:

- Abstand zur Strahlenquelle,
- Aufenthaltsdauer beschränken,
- Abschirmen,
- Abschalten,
- Aufnahme (Inkorporation) verhindern.

Der Gefahrenbereich und somit der Abstand zur Strahlenquelle war in unserem Fall gemäß Feuerwehrdienstvorschrift 500 durch die Fachkräfte auf 50 m festgelegt worden [7]. Innerhalb dieses Bereiches hielten sich nur Einsatzkräfte zu absolut notwendigen Tätigkeiten und so kurz wie möglich auf. Ein Abschalten der Strahlenquelle kam nicht infrage. Eine Inkorporation wurde durch die persönliche Schutzausrüstung verhindert (FFP2-Masken, Untersuchungshandschuhe). Für den Patienten wurde ein Trink‑/Essverbot ausgesprochen – leider war bereits vor Eintreffen der Einsatzkräfte eine Flüssigkeitsaufnahme erfolgt.

Bei Patient 1 lag die Aktivitätsmessung beim Dreifachen der Nullrate, und er galt somit als kontaminiert [7, 8]. In der Regel erfolgt die Dekontamination der entsprechenden Stellen mit Wasser. Der Leitfaden „Der Strahlenunfall“ äußert sich hierzu wie folgt: „Die Haut ist nur an den kontaminierten Stellen mit lauwarmem fließendem Wasser (ca. 30 °C), wie beim normalen Waschen, […] möglichst umgehend zu reinigen“ [2]. Im interdisziplinären Konsens entschied sich die Einsatzleitung jedoch für eine Trockendekontamination, d. h. Abtupfen/Abdrehen mittels Papierhandtuches [4] der betroffenen Stellen.

Aufgrund der Sachkenntnis des Patienten konnte die Dekontamination durch ihn selbst erfolgreich durchgeführt werden [7, 8].

Dies zeigt die Effektivität einfacher Dekontaminationsmaßnahmen auf, die auch mit Mitteln des RD schnell durchgeführt werden können.

Im Nachgang duschte der Patient und wechselte die getragene Kleidung (kein Strahlungsnachweis). Somit war der rettungsdienstliche Einsatz für diesen Patienten nach abschließender negativer Messung und ausführlicher Aufklärung beendet.

Es blieb noch die Möglichkeit der Inkorporation, die aber weder durch eine Messung mittels Teilkörperzähler des nahegelegenen Kernkraftwerks noch durch den Ganzkörperzähler des Bundesamtes für Strahlenschutz (BfS) hätte ausgeschlossen werden können. Trotzdem bestanden bei dem Betroffenen Ängste bezüglich einer möglichen Inkorporation. Aus forensischen Gründen initiierte der durch das Landeskriminalamt hinzugezogene Leiter des regionalen Strahlenschutzzentrums Neuherberg weitergehende, im Ergebnis dann unauffällige Analysen über das Landesamt für Umwelt in Kulmbach.

Wie wichtig das sorgfältige Management eines mutmaßlichen Strahlenunfalls ist, und welche Bedeutung der genauen Kenntnis der Strahlenquelle zukommt, illustriert die Katastrophe von Goiania (Brasilien) im Jahr 1987 [9]: Durch die akzidentelle Bestrahlung und Verbreitung von ca. 80 g (!) Caesiumchlorid (β- und γ‑Strahler) wurden 249 Personen kontaminiert und nahmen über die Jahre mehr als 500 Personen Schaden; nachweislich starben 4 akut, weitere Todesopfer werden damit in Verbindung gebracht. So auch ein 6‑jähriges Kind, welches durch die Inkorporation von Caesium^137^-Staub eine letale Dosis aufnahm. Es handelt sich um einen der weltweit größten Strahlenunfälle außerhalb kerntechnischer Anlagen. Durch Erkennen der radioaktiven Strahlung und Kenntnis der Quelle hätte dieser durch einfache Maßnahmen verhindert werden können.

## Fazit für die Praxis

- Unfälle mit radioaktiver Strahlung sind ein seltenes Ereignis und mit erheblicher Unsicherheit in deren Umgang behaftet. Die Anzahl der eingesetzten Kräfte kann erheblich sein.
- Der Einsatz kann in der Regel mit dem Befolgen einfacher Regeln und dem Tragen der persönlichen Schutzausrüstung, ergänzt um eine Mund-Nasen-Maske entsprechender Schutzklasse (sofern kein umluftunabhängiger Atemschutz notwendig ist, eine FFP3-Maske) und Untersuchungshandschuhe, sicher abgearbeitet werden.
- Selbst vermeintlich einfache Dekontaminationsmaßnahmen, wie das Ablegen der Kleidung und Abtupfen/Abdrehen mit einem Papierhandtuch, sind effektiv und können eine bestehende Kontamination erheblich reduzieren.
- Medizinische Fachberater stehen deutschlandweit in den derzeit 7 regionalen Strahlenschutzzentren als Ansprechpartner rund um die Uhr zur Verfügung und kommen je nach Ereignis auch an die Einsatzstelle. Sie sind eine große Hilfe bei der Bewertung und Einschätzung der medizinischen Lage bzw. Organisation der Patientenversorgung.

## Supplementary Information




