# Supplementary material for: Beautiful stones—And shining faces: An unusual rescue operation
Source: Anaesthesiologie. 2023 May 24;72(6):416–8. [Article in German] doi: 10.1007/s00101-023-01282-4 (PMC10244262; doi:10.1007/s00101-023-01282-4)

Zusatzmaterial zum Beitrag „Schöne Steine – und strahlende Gesichter“ von Ventzke M-M und Kirchinger W (2023) in *Die Anaesthesiologie* .
Beitrag und Zusatzmaterial stehen Ihnen auf www.springermedizin.de zur Verfügung. Bitte geben Sie dort den Beitragstitel in die Suche ein.

Tabelle 1: Ergebnisse der Strahlungsmessung an den Einsatzstellen

**Messort**	**Messwert [µSv/h]**
Anhänger direkt an Plane	1,3
Anhänger in 1 m Abstand	0,22
Fundstücke auf Anhänger 1	2,5
Fundstücke auf Anhänger 2	2,13
Fundstücke auf Anhänger 3	3,77
Wohnung	---

Sv – Sievert

Tabelle 2: Ergebnisse der Strahlungsmessung an beiden Patienten vor und nach Dekontamination

	**Patient 1**	**Patient 2**	**Nullrate**
Erstmessung<br>Gesicht<br>Hände	 90 Bq/cm^2<br>70 Bq/cm^2	keine relevant über die Nullrate hinausgehende Strahlung	30 Bq
nach Dekontamination	keine relevant über die Nullrate hinausgehende Strahlung	entfällt	30 Bq

Bq - Becquerel

Tabelle 3: Kräfteübersicht des Rettungsdienstes und der anderen Fachbereiche

**Rettungsmittel**	**Anzahl**	**Personal**
LNA	1	1/-
OrgL	1	1/-
ELRD	1	1/-
ELW UGSanEL	1	-/3
NEF	1	-/2
RTW	5	-/10
**Summe**		3**/15**
Polizei	3 Streifen	
	Gefahrguttrupp	
	Einsatzzug	
Feuerwehr	Kreisbrandrat	1/-
	Kreisbrandinspektor	1/-
	Kreisbrandmeister	1/-
	Fachberater Gefahrgut	-/2
	CBRN Erkundungs- /Messkomponente	
	CBRN Erkundungs- /Messkomponente	
	DEKON-P-Einheit	
	Löschzug	
Werkfeuerwehr HMGU	Kleinalarmfahrzeug mit Kommandowagen Helmholtz-Zentrum München	1/1
Regionales Strahlenschutzzentrum Neuherberg (RSZ)	RSZ- Einsatzfahrzeug	1/1

LNA – Leitender Notarzt, OrgL – Organisatorischer Leiter, ELRD – Einsatzleiter Rettungsdienst, ELW – Einsatzleitwagen, UGSanEL – Unterstützungsgruppe Sanitätseinsatzleitung, NEF – Notarzteinsatzfahrzeug, RTW – Rettungswagen, CBRN – chemisch, biologisch, radioaktiv, nuklear, DEKON-P – Dekontamination Personen, HMGU - Helmholtz Zentrum München Deutsches Forschungszentrum für Gesundheit und Umwelt, RSZ-Regionales Strahlenschutzzentrum

Abb 1 Trockendekontamination mit Papierhandtüchern

a) exemplarische Kontamination des Unterarmes b) Auflegen von Papierhandtüchern c) Drehen und Abheben der Papierhandtücher, um Kontamination des Unterarmes nicht zu vergrößern wie es beispielsweise durch Reiben der Fall wäre.

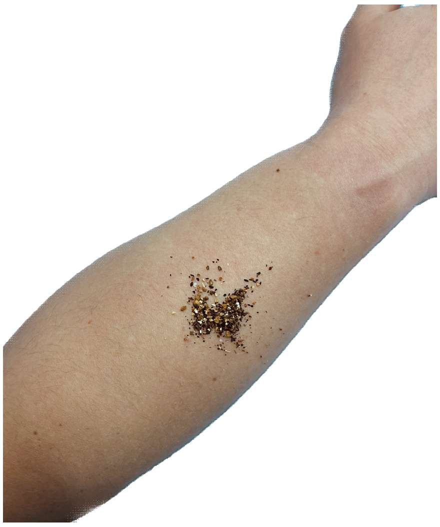

a

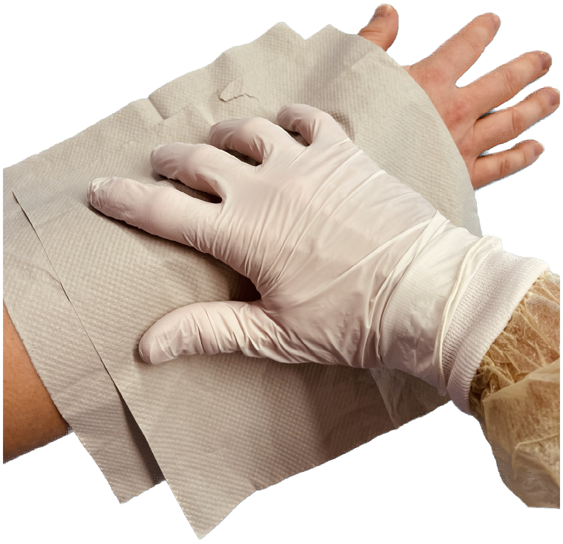

b

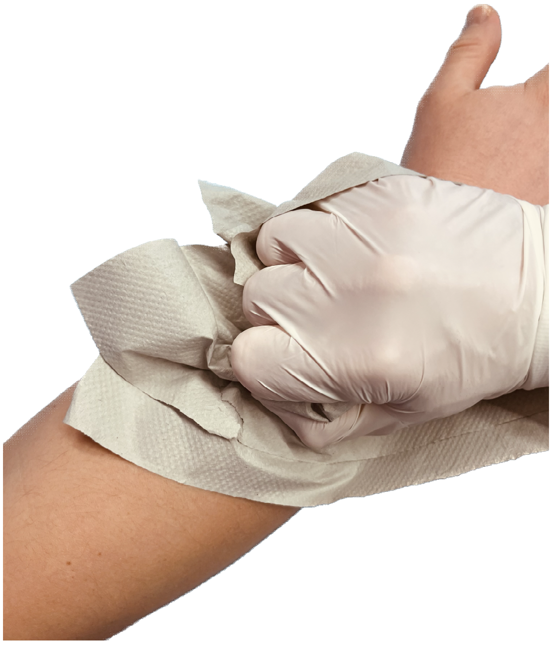

c

Abbildung 2 Verpacken und Verladen des Anhängers durch die Feuerwehr in Zusammenarbeit mit einem beauftragten Transportunternehmen

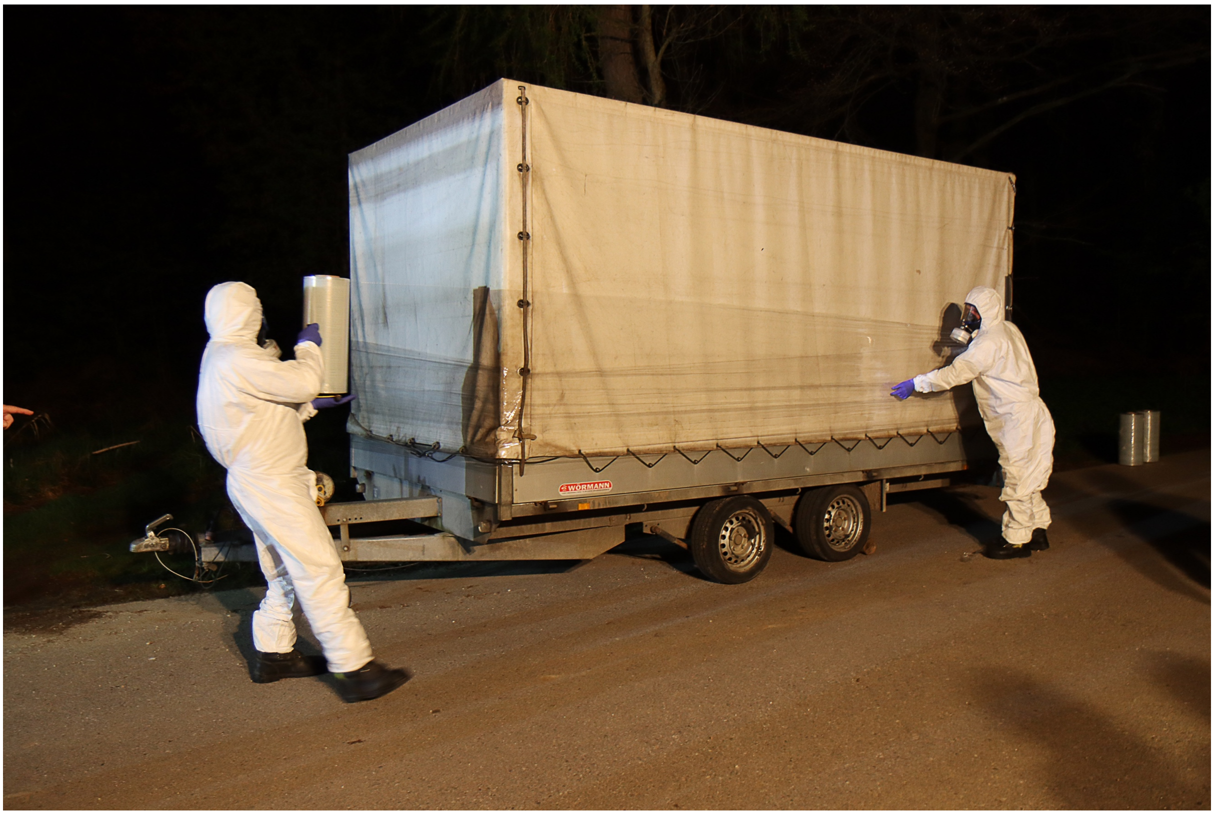

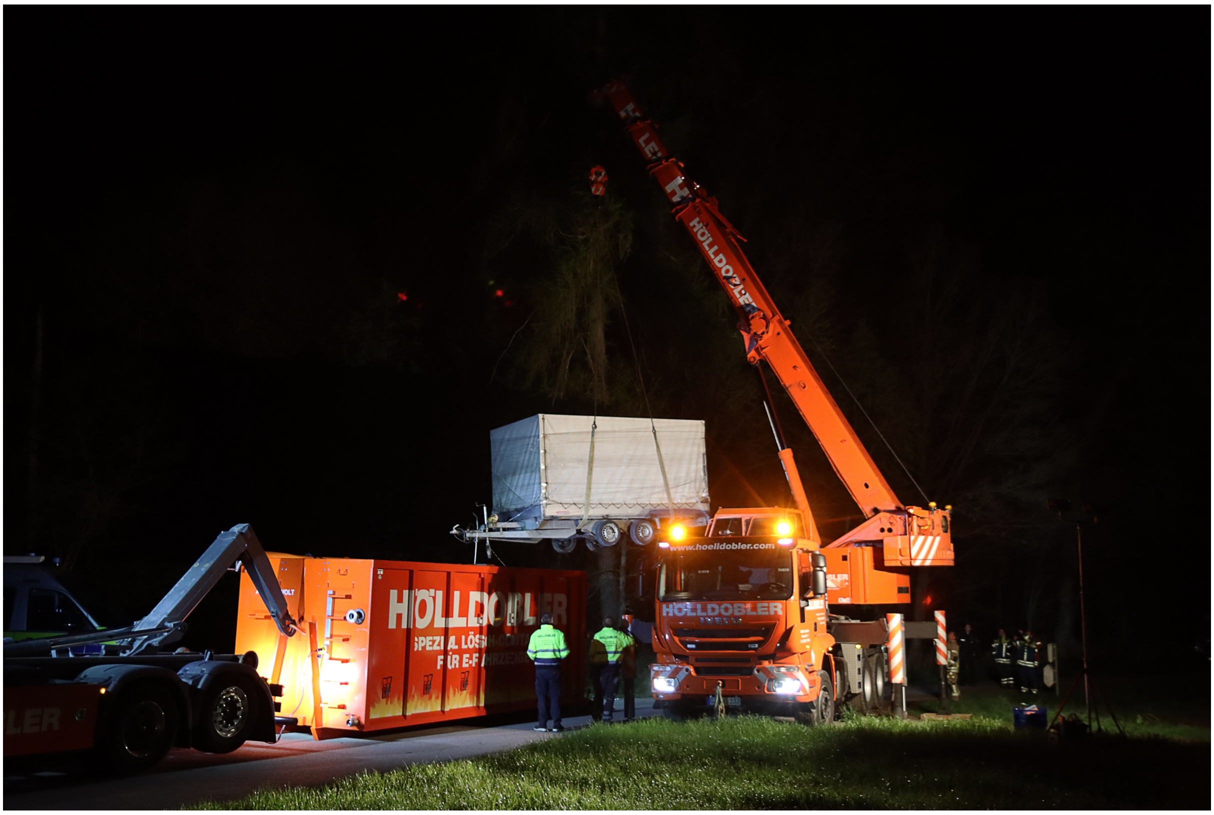

Abbildung 3 Messung durch Kräfte des Regionalen Strahlenschutzzentrums Neuherberg an unterschiedlichen Fundstücken auf dem Anhänger.

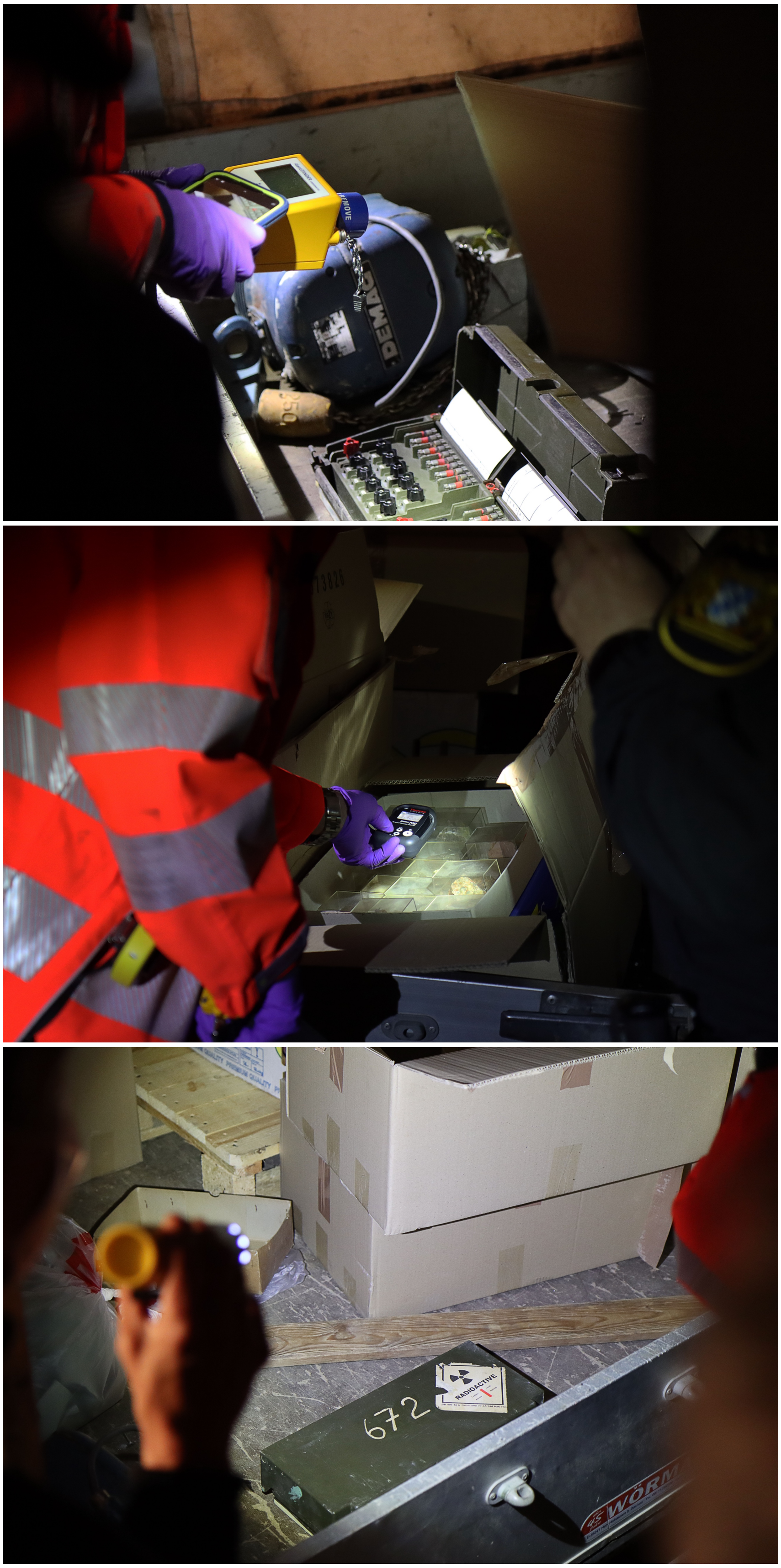

Supplement: Supplementary file 1 [file 101_2023_1282_MOESM1_ESM.pdf]
